# NICE: A Computational Solution to Close the Gap from Colour Perception to Colour Categorization

**DOI:** 10.1371/journal.pone.0149538

**Published:** 2016-03-08

**Authors:** C. Alejandro Parraga, Arash Akbarinia

**Affiliations:** 1 Centre de Visió per Computador (CVC), Barcelona, Spain; 2 Departament de Ciències de la Computació, Universitat Autònoma de Barcelona (UAB), Barcelona, Spain; University College London, UNITED KINGDOM

## Abstract

The segmentation of visible electromagnetic radiation into chromatic categories by the human visual system has been extensively studied from a perceptual point of view, resulting in several colour appearance models. However, there is currently a void when it comes to relate these results to the physiological mechanisms that are known to shape the pre-cortical and cortical visual pathway. This work intends to begin to fill this void by proposing a new physiologically plausible model of colour categorization based on Neural Isoresponsive Colour Ellipsoids (NICE) in the cone-contrast space defined by the main directions of the visual signals entering the visual cortex. The model was adjusted to fit psychophysical measures that concentrate on the categorical boundaries and are consistent with the ellipsoidal isoresponse surfaces of visual cortical neurons. By revealing the shape of such categorical colour regions, our measures allow for a more precise and parsimonious description, connecting well-known early visual processing mechanisms to the less understood phenomenon of colour categorization. To test the feasibility of our method we applied it to exemplary images and a popular ground-truth chart obtaining labelling results that are better than those of current state-of-the-art algorithms.

## Introduction

This study aims at closing the explanatory gap between colour perception and colour categorization by providing a plausible explanation supported by a low-level model (termed Neural Isoresponse Colour Ellipsoids or NICE) that was adjusted to psychophysical results and can be parsimoniously implemented by cortical neurons.

The richly-coloured appearance of the world is in great measure, a creation of our brains. For example, there are no discontinuities in the electromagnetic spectrum of the light reaching us from a rainbow and yet we see hues clearly separated by colour categories. Although the whole process is highly non-linear in nature, the first stages corresponding to low-level colour vision mechanisms are usually represented in a conveniently linear colour space. After the stimulation of the three cone photoreceptors (L, M and S for Long-, Medium- and Short-wavelength sensitive) there is a second stage, partially composed by chromatically opponent combinations of these neural signals at the retinal level. LMS-stimulations are the basis for cone excitation spaces and the responses of opponent and non-opponent cells at the level of the Lateral Geniculate Nucleous (LGN) give birth to various achromatic-tritanopic-deuteranopic spaces or ATD-spaces for short [1–3]. The perceptual metric of these spaces is clearly non-Euclidean, as demonstrated by the shape of discrimination threshold data when plotted in a tristimulus space [4]. Because of these non-linearities, it is not possible to obtain perceptually uniform representations by just applying linear transformations to the photoreceptors’ signals [5] and this is the reason why all perceptually uniform spaces recommended by the regulatory Commission Internationale de l'Eclairage (CIE) such as L*u*v*, L*a*b*, RLab, Llab, etc. involve non-linear transformations. There are also differences between the perceptually-measured cardinal directions (traditionally called “unique hues”) and those predicted by LGN-based models (and measured in cortical neurons) [6–9]. The existence of these alternative cardinal directions suggests that ATD-spaces might have little significance beyond defining an adaptable synapse for the signal entering the visual cortex. To account for these variations, a number of non-linear cortical representations have been proposed which incorporate adaptive processes and other “perceptual” non-linearities that cannot be envisaged as simple basis transformations of the previous stages [10, 11]. Although they give a precise numerical characterization of colours viewed in isolation, none of the physiological models above fully quantify the influence of the illuminant, complex surrounds and complex background on our perception of lightness, brightness, saturation, hue and chroma. These interactions are best quantified by colour appearance models [12], which do not make strong claims about following the different physiological stages found in the human visual system.

As more of the physiology of the primate visual system and in particular the visual cortex becomes understood, the mechanisms that transform cone excitation signals into sensation (colour appearance) are slowly revealed. For example, while LGN cells are hardly susceptible to habituation (i.e. they do not show strong colour appearance aftereffects as a result of prolonged exposure to chromatically modulating stimuli) [13], many neurons in area V1 (the primary visual cortex) are [7]. This and the broadly distributed chromatic preferences of V1 neurons [7, 8, 14–16] suggest that the basis for some psychophysically measured, fundamental chromatic mechanisms may be well established as early as the striate cortex. Indeed, many researchers have looked for a smaller, “specialized” population of neurons likely to be responsible for colour vision in V1. These colour-cells (usually termed “double-opponent”) which respond best to opponent signals from spatially adjacent cones in the retina, have been found to cluster together in “blobs” within V1 [17, 18]. Information from the V1 blobs is then sent to a later visual area called V2, which also contains cells tuned for colour and arranged in “thin stripes”, alternating with cells concerned with other visual attributes such as motion. From there, colour information continues towards other areas in the cortex like V4 and V3, both of them concerned with colour and form. V4 and associated areas contain millimetre-sized clusters called “globs” constituting the first colour processing area to be sensitive to the full range of the colours found in the visible part of the electromagnetic spectrum [19, 20]. There is also growing evidence that the physical location of many neurons in the cortex is also determined by their peak hue sensitivity, with units arranged in partially-overlapping “clusters” [20–22] that respond more to perceptually defined hues than to cone-opponent cardinal directions [23]. More recently, V1 non-linearities have been shown to be partially determined by neurons responding to all colour directions, whose isoresponse surfaces were modelled as ellipsoids in ATD-space with major and minor axes aligned to perceptual cardinal directions [8]. In this context, colour may be represented not by a single brain area, but by spatially distributed activity patterns in the visual cortex clustered according to the colour selectivity of cortical neurons [24].

The gap between our understanding of the neurophysiological machinery of the visual pathway and the actual appearance of colours becomes wider when it comes to categorization, a process by which elements forming a scene are differentiated and grouped, reducing an extremely complex world to cognitively tractable proportions. In the colour domain this reduction is large indeed: from the nearly 2 million colours that can be distinguished perceptually [25] to the nearly 30 categories that can be recalled by a normal subject [26]. Of these, and even smaller subset of 11 categories has been suggested by Berlin and Kay to be common to all cultures and languages with data showing remarkable consistence across culturally diverse populations in the naming of these colour categories [27, 28]. The later results ignited a strong debate in cognitive science about the role of language in perception: one side (the “relativists”, associated to Benjamin Lee Whorf) holding that perception is shaped by the semantic categories of one’s native language, and the other side (the “universalists”) arguing on behalf of a universal underlying beneath thought and perception, which shapes language instead. Scientific consensus has swung between these two sides for a number of years, eventually reaching a compromise position of moderate universality (see review by Kay and Regier [29]) based on evidence which seems to partially support both views: colour terms do affect colour perception while there is indeed a universal background to colour naming. More recent results suggest that language might shape perception primarily in the right visual field which is connected to the brain’s left hemisphere (dominant for language), acting as a sort of partial “linguistic filter” [30–33]. This top-down influence of language is evident in a phenomenon called *categorical perception* (i.e. stimuli located near the categorical boundary are discriminated faster or more accurately than stimuli well within the category) and its effects are likely to disappear when subjects perform a verbally concurrent task [30]. Categorical perception prior to language acquisition is still far from understood, since infants show clear categorical perception effects in their left visual field and none in their right one, suggesting a reversal of hemisphere roles as language is acquired [34]. At the same time, there is growing evidence both from event-related potential (ERP) and functional magnetic resonance imaging (fMRI) studies, that early (low-level) mechanisms also play a large role in the categorical perception of colour. For example, fMRI studies have shown lateralized activation of the language and visual areas when subjects were asked to discriminate colours of different lexical categories, suggesting that the language area might act as a top-down modulator of the visual cortex [35]. Regardless of this intimate relationship between colour categorization and language, studies of non-verbal categorization using colour-sorting tasks [36, 37], free categorization tasks [38], visual search [39] and infant’s eye movements [40] have shown that there must be some kind of intermediate, free-from-language colour categorization stage. In particular, psychophysical experiments [38] revealed that subjects can perform categorization tasks without colour terms (i.e. on the same stimulus used for low-level colour perception tasks) and moreover, they can perform discrimination tasks without any evidence of categorical boundary effects. The exact site of this intermediate stage of colour sensation prior to learned colour-naming is unknown, although there is growing physiological and neuroimaging evidence (from chromatic selectivity studies [41], effects of luminance contrast [42], universal colour naming constraints [43], hue maps [21], chromatic motion discrimination [44], and the effects of V2 lesions [45]) pointing out to V1. However, the precise neural mechanisms behind this complex partitioning of chromatic space are still largely unknown. This work aims at closing the gap between what is known about the physiology of the visual system and current pragmatic solutions to the colour categorization problem. To do this we performed a psychophysical experiment to specifically measure the boundaries of colour categorical perception in the colour space likely to represent the signals entering the visual cortex. From these measures we estimated the kinds of mathematical operations necessary to define those categorical regions, which may result from the overall effect of weighted excitatory and inhibitory responses over a net of synaptic connections in the brain. If these operations are implemented in V1, the most likely candidates to perform such low-level segmentation of chromatic space are neurons similar to neuron type 3 described by Horwitz and Hass [8].

### Observer variability in colour naming

Precisely measuring the boundaries of chromatic categorization is a difficult problem, usually substituted by the simpler problem of determining chromatic regions whose names observers agree upon. However, this is still a complex task, made difficult by the large individual differences (even among speakers of the same language) in selecting the best examples of each colour also called “focal colours”. Indeed, focal colours selected by speakers of different languages are sometimes more similar than those selected by speakers of the same language. Although this phenomenon has been extensively studied in the context of colour appearance and unique hues [46–49], the precise source of these individual differences remains unknown [50]. The most obvious explanation is perhaps differences in the physiology, e.g. large differences in macular and lens densities across individuals [50–52], resulting in a variable shift away from shorter wavelengths, which are absorbed by the ocular media. Pigmentation density also increases with age, although no significant difference in colour judgements with age has been found [53, 54]. Cone photopigments are also subject to considerable variations both in the peak wavelength and bandwidth of their spectral sensitivity because of polymorphisms in the encoding genes [55] and variations in pigment density [56]. Variations in the ratio of L:M cones are also large for individual subjects (from less than 1 up to one order of magnitude in some cases) [57], in fact they are far too large to account for individual differences in unique hues [58, 59]. Pronounced spectral sensitivity differences do indeed play a role in the placement of unique hues: red-green anomalous trichromats for example, place unique yellow at significantly different wavelengths than normals, exposing the limits to whatever compensatory neural mechanism the visual system may have to balance physiological variations [60]. Some authors [61–64] have argued that environmental factors may account for the mismatch between the appearance of colours and observers’ chromatic sensitivity [59]. For example, a neural adjustment based on the average illuminant of the environment may account for the independence of unique yellow (the point where the output of the red-green opponent system is minimal) from different cone ratios, small L and M sensitivity differences or the effects of aging. If this was true, then different natural environments (i.e. different natural statistics) with either geographical or seasonal variations would influence colour appearance and naming measures [65]. This hypothesis is complementary to related arguments pointing out the importance of ripening fruits and foliage in shaping the characteristics of primate trichromatic colour vision [66–68], thus the special salience of red in human languages [27]. In a more recent study, Witzel and Gegenfurtner [69] measured both sensitivity to colour differences and categorical boundaries, concluding that although there is a link between them, sensitivity to colour is not inherently determined by categorization abilities. There is also an argument to be made about the contribution of linguistic/cultural contexts to subjective colour judgements [70, 71]. Webster *et al* [72] tested colour name variations in two distinct groups (US and Indian observers) and concluded that basic colour terms were similar, with modest variations in their loci across different populations. Further studies based on the World Color Survey database [50, 73] concluded that despite individual differences in focal colours, there were strong universal tendencies, giving support to the original Berlin and Kay hypothesis [27].

### Measuring categorical boundaries in colour space

Given these difficulties, it is not surprising that much of the effort to study chromatic categorization focuses in its regularities. Psychophysical studies have proposed the existence of “colour consensus areas” [74, 75] emphasizing the regularities of colour naming space [76] through paradigms where subjects had to name coloured chips or cards whose colour was generally sampled around regions of suspected consensus [74]. These psychophysical results form the basis of computational colour-category models such as that of Benavente *et al* [77] which assumes that the boundaries are somehow equidistant from the focal colours and their properties (position, slope, etc.) are arbitrarily imposed or interpolated. The same applies to the transition that occurs when a colour loses its saturation, becoming “grey” (e.g. the centre of most colour spaces). Although less common, measures of the boundary regions have been included in some categorical modelling work [78] and in work exploring the relationship between colour categories and colour constancy [79, 80]. In the last example, measurements were part of an evenly-spaced sampling of the colour space without particular attention on the actual borders. It is remarkable that although categorical boundaries are the most crucial part of any colour-category model (this is where the uncertainty is bigger and thus data collection is most critical), almost all modelling is arguably based on the precise colours considered by speakers of many languages as “focal” and their variations [64, 78, 81–87].

In this work we performed a psychophysical experiment to measure the boundaries of nine universal chromatic categories (form the eleven originally proposed by Berlin and Kay) in 3-dimensional (3D) colour space. These boundaries clearly define the shapes of nine 3D ellipsoids when plotted in the ATD-type colour space determined by the colour opponent signals that arrive at the visual cortex from the LGN. Following this initial discovery, we present a model for decoding ATD-type colour opponent signals and constructing a set of universal chromatic categories that attempts to relate its internal mechanics to what it is known about the underlying human physiology, in particular the operation of V1 neurons in the striate cortex [8]. Our psychophysical experiment is particularly relevant since (a) by collecting more data points in the categorical boundaries, it provides a more veridical picture of within-observer variability, which in our case happened to be larger than the variability across observers; (b) it takes into account the effects of local contrast such as the influence of coloured backgrounds in perceived saturation; (c) it accounts for the role of lightness, thus adding the third dimension to colour categorisation; (d) it allows to link a seemingly wide range of colour processing phenomena to colour naming; and (e) it shows the analogy between our ellipsoid model and the responsiveness of cells in V1. By proposing a low-level model of chromatic categorization which is based on psychophysical data, and whose algorithm can be plausibly implemented by cortical neurons we expect to provide a link between cortical physiology and current pragmatic solutions to the colour categorization problem.

The overall organization of this paper is as follows: in the next section (*Methods*) we describe our psychophysical experiment. In the *Results* section we reveal the intrinsic 3D-shape of these regions when plotted in the chromatically-opponent LGN-based space, propose a mathematical model and test it against ground-truth images. In the *Discussion* section we analyse how our model relates to previous models in the literature and whether these 3D regions could be parsimoniously generated by aggregating the output of V1 neurons.

## Materials and Methods

In this section we describe the psychophysical method used for measuring the boundary regions between pairs of colour categories, which is designed to collect most data points where they are most needed, thus countering any informational unbalance.

### A psychophysical experiment designed to measure colour boundary regions

**Fig 1:**
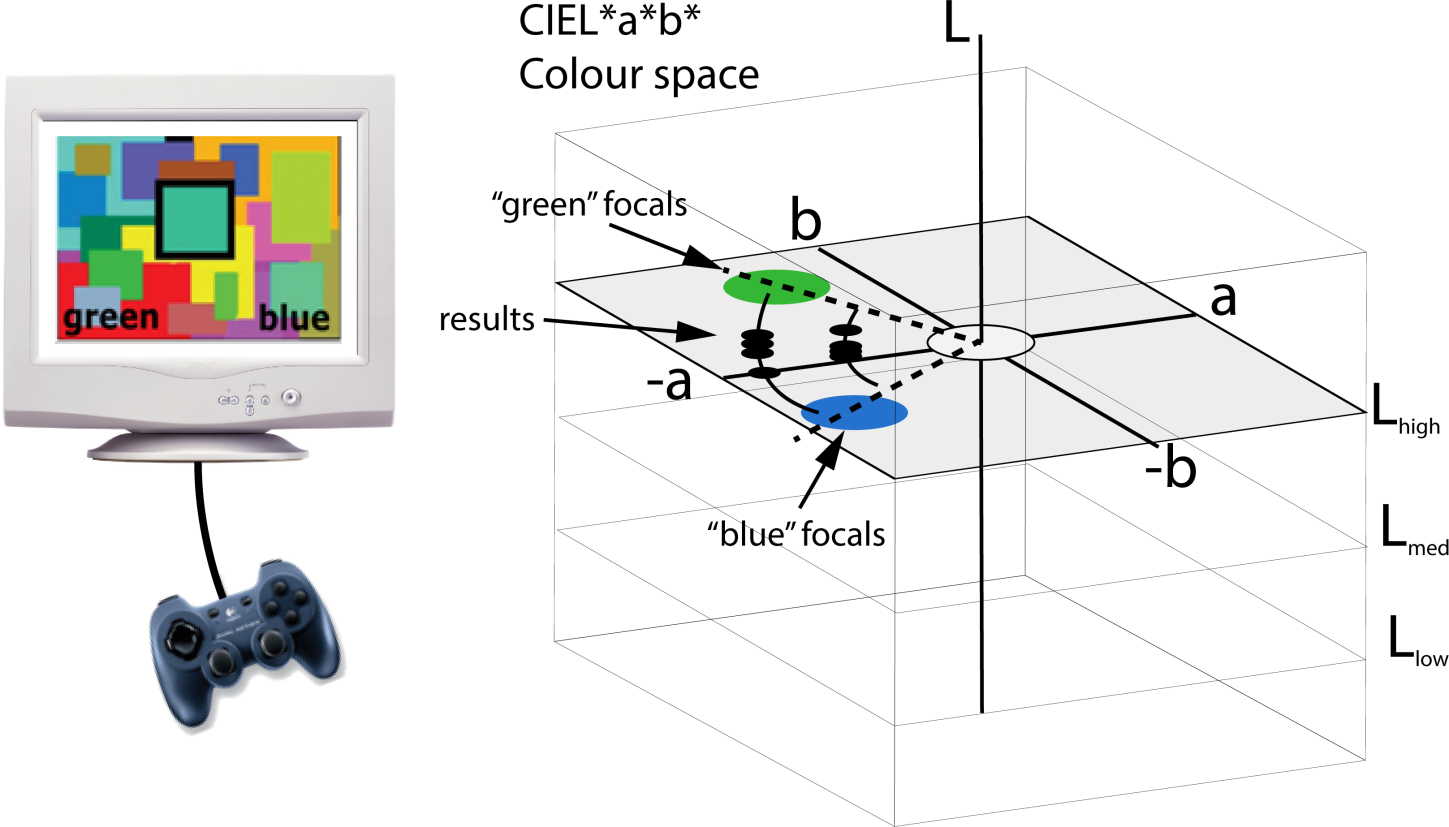
Schematics of the experiment. Subjects manipulated the hue of a central test patch using a gamepad (left). The test patch was embedded inside a coloured Mondrian (Condition 1) or a mid-lightness grey background (Condition 2). Two colour names were presented at the bottom of the screen and subjects had to produce colours equally distant from those represented by the names. They were only allowed to modify the colour along predetermined paths (e.g. lines or concentric arches of randomised radius in CIE L*a*b* space), which started and ended well inside the consensus colour-name regions corresponding to the colour names on the screen. There were no time or head movement constraints.

Because of the large intra-and inter-subject variability of chromatic categorization, we decided to measure chromatic boundaries by means of the *method of adjustments* [88] which has been used before in the literature [89]. This method maximises the number of data points and the speed of data collection, reducing tediousness by allowing observers to keep control over the stimulus therefore improving overall subject performance. In a typical experiment, subjects sit inside a dark room in front of a monitor and are presented with two colour name words written in English at the bottom of the screen and a coloured square (test patch) outlined by thick black lines at the centre (see left panel in Fig 1). They are asked to manipulate the chromaticity of the central test patch using a gamepad until they find a colour that is “midway between the colours written at the bottom”. The colour names written below were always pairs of neighbouring colour categories in CIE L*a*b* space (see right panel in Fig 1), selected from the set of 11 basic colour terms originally proposed by Berlin and Kay [27]. We chose to operate in CIE L*a*b* colour space because it provides a good approximation to perceptually uniform movement steps (for a review on the advantages and disadvantages of CIE L*a*b* see [90]).

The experimental design allowed subjects to alter the chromaticity of the test patch along fixed pathways belonging to six constant lightness planes in CIE L*a*b* space. Two types of pathways were used: constant-saturation *arch pathways* (see Fig 1) and *linear pathways* where both hue and saturation were simultaneously modified. Linear pathways were used when it was not possible to connect two well defined chromatic regions using arch pathways. Each pathway was always contained within one of six different constant-lightness planes. In the case of arch pathways, the distance from any given arch to the centre of CIE L*a*b* space (the radius) was a random number larger than 10 CIE L*a*b* units, and both extremes of the arch were set on unambiguous focal colour regions, obtained from the model of Benavente *et al* [81]. The actual maximum value of the radius depended on the monitor’s colour gamut, and was different for the lightness levels considered. Line pathways were simply random lines connecting unambiguous focal colour regions.

To measure the boundaries of Grey with all other colours, subjects were allowed to alter only the saturation of the test patch along radial pathways of constant-hue, stemming out from the centre of CIE L*a*b* space. The angular values (hues) of these pathways were randomised. The experiment was conducted in six lightness planes with L = [36, 47, 58, 76, 81, 86] lightness units, three of them (36, 58 and 81) chosen to be the same as in our previous experiments [78]. The other lightness levels (47, 76, 86) were chosen out of convenience to target specific boundaries.

To avoid errors of expectation and habituation the starting points of all runs were randomised along a given pathway. To make it impossible for subjects to simply “count button clicks”, we reversed the direction of movement once the observer reached the end of a pathway. For example, in the experiment shown in Fig 1 our subject would press left/right buttons to turn the test patch greener or bluer and upon reaching the end of the arch pathway, the patch would reverse its colour turning towards the opposite category, making it difficult to find the precise “ends” of the pathways. Button presses were set to produce steps of one CIE L*a*b* unit (ΔE*).

To provide a common reference for lightness, a 23 mm wide white frame (D65, 124 cd/m^2^) outlined the border of the screen and a 10 mm black frame outlined the border of the test patch in all cases. Both experiments were run under two conditions, *Condition 1* and *Condition 2*.

In *Condition 1*, the test patch was overlaid on top of a coloured Mondrian. This Mondrian was dynamically created for each run by selecting a random sample of 200 colours from within the CIE L*a*b* range of L[0, 100] a[-60, 60] and b[-60, 60]. They were chosen so that their mean chromaticity was mid-luminance grey (Lab = [50, 0, 0]). Each Mondrian was unique. In *Condition 2*, the test patch was presented on top of a mid-grey (D65, 62 cd/m^2^) background.

The experiment was run on a calibrated cathode ray tube (CRT) monitor (Viewsonic pf227f) controlled by a Cambridge Research Systems (CRS Ltd) ViSaGe Visual Stimulus Generator capable of providing 14-bit colour per channel. The calibration was made using customary CRS Ltd software and a ColorCal (Minolta) colorimeter. Subjects selected colours by pressing buttons on a Logitech gamepad. Central test patches (squares) subtended 5.2 deg to the observers at a viewing distance of 156 cm and the presentation time was unlimited, although subjects were encouraged to spend no more than 30 seconds per trial. Trials were presented at 5 second intervals. Viewing was binocular and unconstrained. The room was completely dark (the walls were lined in black and the monitor was the only light source). An experimental session consisted of a random selection of the 51 chromatic boundaries shown in of Table 1 (10 trials for each boundary).

**Table 1:** Summary of the chromatic boundaries explored in both experiments. Column “L” indicates the lightness value of each given CIE L*a*b* plane considered, column “Boundaries” indicates the chromatic boundary considered and column “Path” indicates the type of path followed by the experimental algorithm. “A” represents an “arch” of equal saturation and “L” represents an arbitrarily defined “line”.

A–Chromatic Boundaries			B–Achromatic Boundaries		
L	Boundary	Path	L	Boundary	Path
36	Red-Brown (R-Br)	L	36	Grey-Green (Gr-G)	L
36	Brown-Green (Br-G)	A	36	Grey-Blue (Gr-B)	L
36	Green-Blue (G-B)	A	36	Grey-Purple (Gr-Pu)	L
36	Blue-Purple (B-Pp)	A	36	Grey-Red (Gr-R)	L
36	Purple-Red (Pp-R)	A	36	Grey-Brown (Gr-Br)	L
47	Red-Brown (R-Br)	L	47	Grey-Red (G-R)	L
47	Brown-Green (Br-G)	A	47	Grey-Brown (G-Br)	L
47	Purple-Red (Gr-B)	A			
58	Pink-Red (Pk-R)	A	58	Grey-Green (Gr-G)	L
58	Red-Orange (R-O)	L	58	Grey-Blue (Gr-B)	L
58	Orange-Brown (O-Br)	L	58	Grey-Purple (Gr-Pu)	L
58	Brown-Green (Br-G)	A	58	Grey-Pink (Gr-Pk)	L
58	Green-Blue (G-B)	A	58	Grey-Red (Gr-R)	L
58	Blue-Purple (B-Pp)	A	58	Grey-Orange (Gr-O)	L
58	Purple-Pink (Pu-Pk)	A	58	Grey-Yellow (Gr-Y)	L
			58	Grey-Brown (Gr-Br)	L
76	Pink-Orange (Pk-O)	A	76	Grey-Pink (G-Pk)	L
76	Orange-Yellow (O-Y)	L	76	Grey-Orange (G-O)	L
76	Yellow-Green (Y-G)	L			
76	Purple-Pink (Pp-Pk)	A			
81	Pink-Orange (Pk-O)	A	81	Grey-Green (Gr-G)	L
81	Orange-Yellow (O-Y)	L	81	Grey-Blue (Gr-B)	L
81	Yellow-Green (Y-G)	L	81	Grey-Purple (Gr-Pu)	L
81	Green-Blue (G-B)	A	81	Grey-Pink (Gr-Pk)	L
81	Blue-Purple (B-Pp)	A	81	Grey-Orange (Gr-O)	L
81	Purple-Pink (Pu-Pk)	A	81	Grey-Yellow (Gr-Y)	L
86	Orange-Yellow (O-Y)	L	86	Grey-Orange (G-O)	L
86	Yellow-Green (Y-G)	L	86	Grey-Yellow (G-Y)	L

We recruited 17 paid subjects (11 females and 6 males between 18 and 30 years old) among the exchange student and the local resident population. They were all native English speakers, had normal or corrected-to-normal visual acuity and their colour vision was tested using the Ishihara and the Farnsworth D-15 colour tests. Subjects were encouraged to take long breaks between experimental sessions. Experimental sessions lasted between 40 and 60 minutes depending on individual observers. Oral consent was obtained from the participants. No written consent was necessary since the data were analysed anonymously.

Boundaries were measured on constant-lightness planes (no measurements were made using lightness-only, vertical pathways). We also analysed whether these boundaries are different for chromatic and achromatic background (Conditions 1 and 2). The current paradigm was decided after discarding our previous yes/no staircase paradigm [91] which proved to be too precise (apart from slow and tedious) for the task proposed here. By using this particular paradigm we sacrificed experimental precision in favour of much larger amounts of data, in a procedure that is more consistent with the large variability of the subject’s responses obtained here (see below).

In summary, our paradigm targeted the boundaries of the focal colour regions proposed by Berlin and Kay including achromatic/chromatic boundaries (Grey versus all other colours). The selection of pathways and boundaries was made considering the minimum amount of points to define a curve in a plane and the 3-dimensional shape and position of categorical regions estimated by other authors [78, 79, 81]. Not all subjects participated in all pathways measurements.

## Results

In this section we present our psychophysical results and fit them with a parametric model based on the ellipsoidal isoresponse surfaces of visual cortical neurons. To test the feasibility of our method we applied it to exemplary images and a popular ground-truth chart.

### Psychophysical results

**Fig 2:**
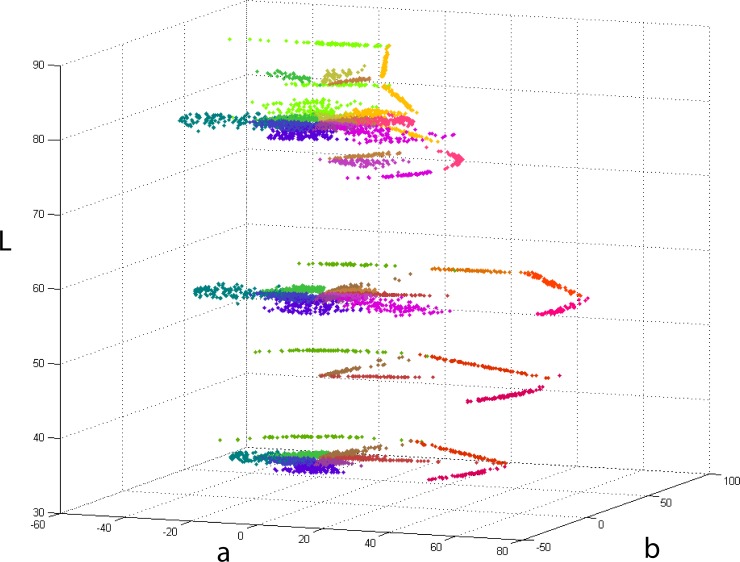
Results plotted in CIE L*a*b*. Coloured points represent individual trials for 17 participants (all native English speakers). Each colour corresponds to a hue-dependent boundary. For clarity, all colours in the plot were calculated as the average of the two focals that determine the boundary. For example, points that correspond to the boundary between Green and Blue (G-B) are coloured turquoise, which is the mathematical average resulting from the combination of these colours. The reference white was that of the frame described in the methods section.

Fig 2 shows our results in CIE L*a*b* space, plotted of all six L-levels considered. Coloured dots represent the boundary locations selected by subjects in individual trials in both conditions. For representational purposes, pixel colours in the plot were obtained by averaging the two focal colours involved in each boundary experiment. The “white” locus in all plots (L = 100, a = 0, b = 0) corresponds to the white frame (D65, 124 cd/m^2^) present in all experiments as described above.

Most of the measurements in Fig 2 were made at three lightness levels (L = [36, 58, 81]) and concentrated on boundaries of relatively low saturation (near the achromatic locus). They were made either following constant lightness and saturation (arch) pathways or constant lightness and hue (radial) pathways in the case of Grey versus all other colours. Here subjects manipulated the central stimulus patch in the saturation dimension of CIE L*a*b* only. The rest of the measures were obtained by following arbitrary paths designed to complement the radial and arch pathways. The main reason for this was the asymmetric nature of the monitor gamut in CIEL*a*b* space which limited in practice the number of options available for constant-saturation pathways between pairs of highly saturated colours. Arbitrary pathways measures were located in regions of high saturation (near the edges of the monitor’s gamut), were not restricted by saturation or hue and followed straight lines of constant lightness. Columns “path” in Table 1 indicate the type of pathway used in each case. The choice of lightness levels was determined by the need to fit the curvature of our categorical model along the lightness dimension, considering that to fit any conic section curve in two dimensions once the centre is determined, a minimum of 3 non-collinear points is necessary [92]. This is the reason why boundaries were measured along three extra lightness planes (L = [47, 76, 86]) allowing us to fit curved 3D surfaces to all of the 9 categories.

**Fig 3:**
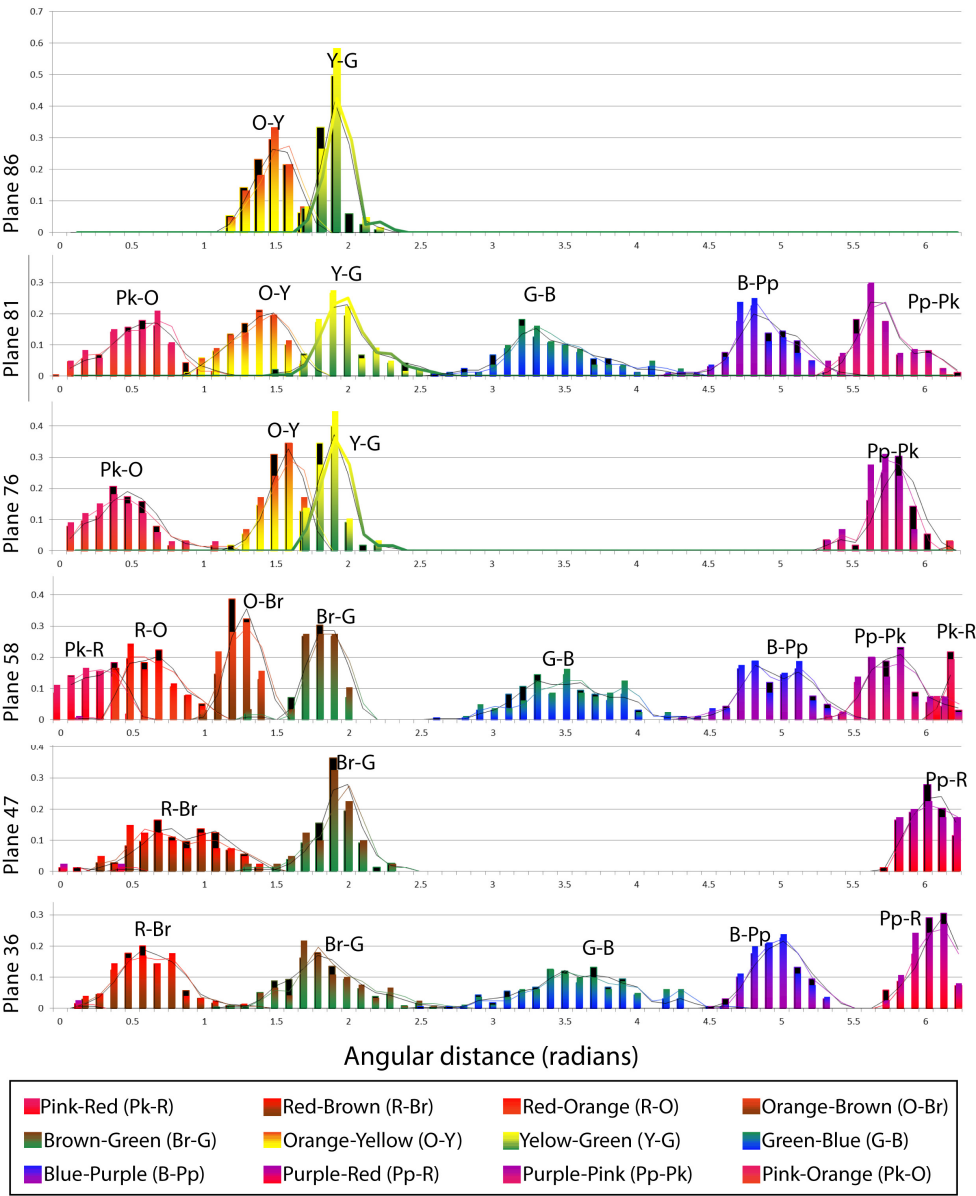
Histograms of the boundaries plotted in Fig 2. The same data was plotted as histograms whose independent variable was the angle (hue). Each of the horizontal panels corresponds to one of the lightness levels tested and the x-axis corresponds to the angular distance in radians measured counterclockwise from the horizontal. Coloured bars represent Condition 1 (chromatic background) results and black bars underneath represent Condition 2 (achromatic background) results. The curves show the rolling average applied to the histogram data (coloured for Condition 1 and black for Condition 2) which has the effect of smoothing out local variability.

Fig 3 shows the histogram distributions of each measured chromatic category boundary. The coloured histogram bars correspond to data collected under Condition 1 and the black bars correspond to data collected under Condition 2. The x-axis in Fig 3 is the angle (in radians) which determines the hue in CIE L*a*b*, measured counterclockwise around the centre with the horizontal as zero. The y-axis represents the fraction of trials belonging to each angular interval (bins). We have superimposed the curves corresponding to the rolling averages to show the differences between both sets of data. Coloured curves correspond to Condition 1 and black curves correspond to Condition 2.

**Fig 4:**
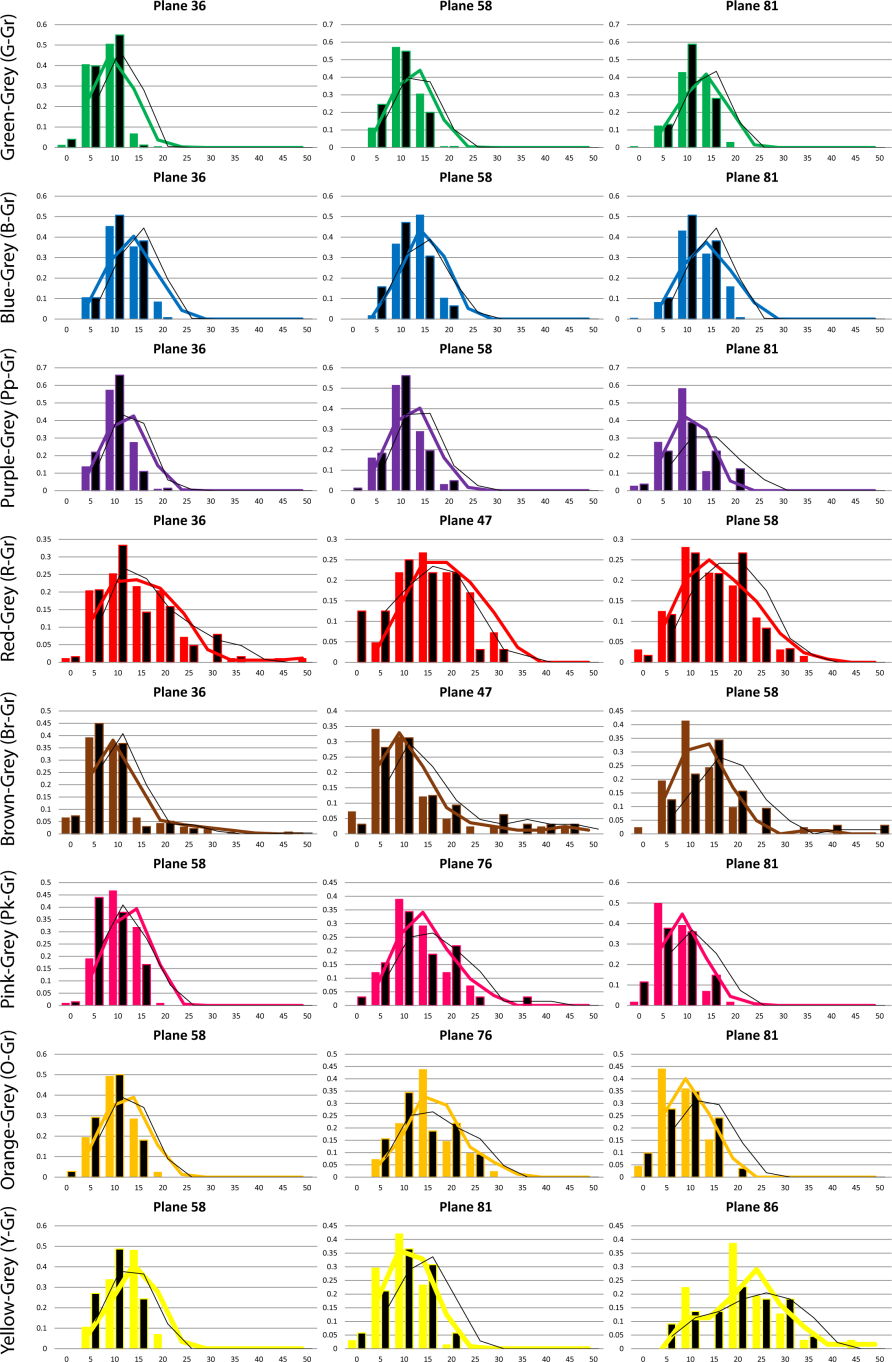
Details of the Grey-versus-other-colours boundaries. The data was plotted as histograms where the abscissa represents the distance to the achromatic locus (saturation). Plots are arranged in rows and columns, where each of the columns corresponds to one of the three lightness levels tested. The rows correspond to a particular boundary between Grey and other colour regions. Coloured bars represent Condition 1 (chromatic background) and black bars represent Condition 2 (achromatic background). The curves show the rolling average of the data (coloured for Condition 1 and black for Condition 2).

Although the variance of surrounding colour has been shown to have an effect in the perceived saturation (not the hue) of embedded patches [93], the shape and position of the lines in Fig 3 show no evidence of systematic effects of the type of background (Conditions 1 and 2) in our categorical hue boundaries. The choice of a variegated background that randomly changed in shape and hue for every trial was deliberately imposed to neutralize possible chromatic induction effects [94, 95] in the long term, by averaging their influence. We also explored the effects of background colour variance on our saturation-only boundaries. Fig 4 shows the histograms of all results involving the achromatic categories (Grey versus other colours). Each of these boundaries was tested in at least 3 lightness levels (shown in different columns in Fig 4). Coloured bars represent experiments with coloured backgrounds (Condition 1) and black bars represent experiments with achromatic background (Condition 2). Our results show no influence of the type of background, and this was confirmed by running paired Student’s T-tests (p>0.05) for the two conditions in all hue and saturation boundaries. We hypothesized that these saturation effects are generally small and our method (which aggregates the data points of many observers in a single histogram) lacks the precision to distinguish between them. Another possible explanation is that the black lines surrounding the central patch in Fig 1 (whose purpose is to reduce the local influence of the background) may have contributed to reduce the overall variance effect. Since we could not find any degree of influence of the type of background on our experimental results, we decided to aggregate all experimental data irrespective of condition in our subsequent modelling. The rationale here is that a general model of categorization will benefit from having a larger number of data points to calculate averages, i.e. including rather than excluding inter- and intra-subject variation.

A closer inspection of the histograms in Fig 3 reveals some possible binomial distributions in the Green-Blue (G-B) and Blue-Purple (B-Pp) boundaries, in the mid-lightness level plane (L = 58). This might be caused by the presence of extra colour categories (e.g. turquoise) which was not considered in the original experiment. The emergence of turquoise has also been confirmed in a previous experiment [78] where psychophysical results using 2-alternative forced choice methods showed binomial results distributions in the B-G boundary. Although our methods were designed to test for the boundaries of the 11 universal colour categories proposed by Berlin and Kay, there is no assumption regarding the presence of other (non-explicit) categories in the data.

Since we are attempting to bridge the gap between the subjective phenomenon of chromatic categorization and the low level mechanisms based on the responses of V1 neurons, it is important to consider the sources of error inherent to the process from both psychological and neural sources. The former is manifest in the variability of the responses of a subject against the whole subject population and the latter is evidenced in the variability of responses an observer gives when presented with the same task many times. Table 2 shows estimations of the inter- and intra- observer variability of our results. Inter-observer variability indicates the extent to which individual observers agree with the average observer whereas intra-observer variability indicates how consistent individual observers are across different trials. Both measures were calculated in CIEL*a*b* colour space (in *ΔE* units, which represent *jnd*s) and were averaged for all observers. Given that the trajectories were different depending on the boundary considered (i.e. there were “lines” and “arches”, see Table 1) our measurements were obtained as follows. In the case of “lines” we considered Euclidean distances from the mean value of the results. In the case of “arches”, first we projected all trajectories to the mean curved path and converted this path to a line, after this we proceeded as before. Results in Table 2 show that variability was generally smaller in the achromatic-chromatic boundaries. Also, in most cases intra-observer variability was larger than inter-observer: in average data collected over the same observer was 1.3 times further away from the mean than data pooled across all observers. In other words, pooling over more subjects reduced the standard deviation with respect to having one subject repeating the same experiment many times. This trend was reversed in some low-lightness boundaries (Br-G and Pp-R for L = 36 and L = 47).

**Table 2:** Consistency between and within subjects for each measured boundary. The table on the left shows the measures obtained for chromatic boundaries and the table on the right for achromatic (Gr)-to-other-colours boundaries. Inter-observer variabilities were obtained by calculating the standard deviation of each observer from the mean (all observers). Similarly, intra-observer variabilities were obtained by calculating the standard deviation of each observer from its own mean (since each observer repeated the same experiment many times). Units correspond to CIE L*a*b* units (equivalent to a *jnd*).

A – Chromatic Boundaries				B – Achromatic boundaries			
L	Boundary	inter	Intra	L	Boundary		intra
36	Red-Brown	10.43	11.22	36	Grey-Green	1.38	4.77
36	Brown-Green	8.13	6.96	36	Grey-Blue	2.09	5.56
36	Green-Blue	4.12	4.75	36	Grey-Purple	1.14	4.02
36	Blue-Purple	2.68	3.28	36	Grey-Red	4.95	7.13
36	Purple-Red	6.77	6.24	36	Grey-Brown	7.17	7.23
47	Red-Brown	11.31	11.49	47	Grey-Red	5.49	5.43
47	Brown-Green	8.48	8.27	47	Grey-Brown	6.29	6.73
47	Purple-Red	7.68	7.37	58	Grey-Green	1.36	5.30
58	Pink-Red	5.54	7.98	58	Grey-Blue	1.72	6.62
58	Red-Orange	6.29	12.66	58	Grey-Purple	2.38	3.88
58	Orange-Brown	4.23	4.87	58	Grey-Pink	2.88	3.54
58	Brown-Green	7.67	7.86	58	Grey-Red	4.35	5.31
58	Green-Blue	4.45	6.32	58	Grey-Orange	2.56	3.43
58	Blue-Purple	4.08	4.30	58	Grey-Yellow	1.80	3.83
58	Purple-Pink	4.41	4.99	58	Grey-Brown	4.27	5.72
76	Pink-Orange	4.83	9.10	76	Grey-Pink	2.88	6.09
76	Orange-Yellow	5.86	7.88	76	Grey-Orange	4.75	4.16
76	Yellow-Green	4.87	5.13				
76	Purple-Pink	4.41	4.49				
81	Pink-Orange	2.80	4.97	81	Grey-Green	2.05	4.89
81	Orange-Yellow	8.83	10.04	81	Grey-Blue	2.58	6.44
81	Yellow-Green	5.12	6.11	81	Grey-Purple	2.77	4.09
81	Green-Blue	4.52	6.87	81	Grey-Pink	3.06	3.04
81	Blue-Purple	3.14	4.57	81	Grey-Orange	3.55	4.19
81	Purple-Pink	2.83	5.06	81	Grey-Yellow	3.10	3.77
86	Orange-Yellow	8.24	11.21	86	Grey-Orange	3.43	3.41
86	Yellow-Green	3.83	6.13	86	Grey-Yellow	5.40	5.41

A few boundaries (R-Br at low-lightness, R-O at mid-lightness and O-Y at high-lightness) produced larger variabilities than the rest (StDev >10 jnd). However, in all these cases aggregating the results of many observers reduced the standard deviation. Achromatic-to-chromatic boundaries produced much more consistent results (average inter- and intra-observer StDevs equal to 3.34 and 4.96 respectively) indicating smaller “neural” and “psychological” variability.

### Modelling results

Once the categorical boundaries have been measured, we proceeded to look for a suitable colour space where to describe them mathematically. Since our general objective is to link appearance results to the colour machinery of the human visual system, we searched for the most likely representation of the same data allowed by the physiological constraints of visual neurons. Following this, we fitted the data to a collection of isoresponsive ellipsoids, therefore we named our model NICE (Neural Isoresponse Colour Ellipsoids).

### Linking colour appearance to L, M, S cone excitations

Signals from the cones undergo an important normalization before reaching later visual processing stages. This normalization, which is likely to happen at the level of the retina, converts cone excitations to a contrast representation. The first step in explaining our colour appearance results in terms of physiological mechanisms is to choose a convenient physiologically-based ATD-representation that combines and normalizes the outputs from the three cone-photoreceptors in the retina. There are many equiluminant planes suitable for this purpose, such as those proposed by Luther [96], MacLeod and Boynton [97, 98] and the derivative proposed by Smith and Pokorny [99]. These cone chromaticity spaces, which represent chromatic data in terms of linear transformations of the cone excitations, consist of a reciprocal L and M-excitation axis and a normalized S-excitation axis. In our case, we used the cone fundamentals proposed by Smith and Pokorny, which were calculated from the CIE 1931 2-deg colour matching functions as revised by Judd and Vos [100]. Since luminance information is removed from chromaticity planes, we added a third “Y” axis to represent the different lightness levels of our data. The values represented in this Y-axis are obtained by adding L + M cone excitations, as the exact shape of the luminous efficiency function is obtained from adding the L to the M spectral sensitivities [101]. We call this space *lsY* and to convert our psychophysical results from CIE L*a*b* to this new space we first converted them to CIE XYZ using the standard set of equations [12] considering the monitor’s D65 as the reference white and later transformed them to LMS cone excitations using the following transformation matrix [99]:

$$\left(\begin{array}{c} L\\ M\\ S \end{array}\right)=\left(\begin{array}{ccc} 0.15516 & 0.54307 & -0.03287\\ -0.15516 & 0.45692 & 0.03287\\ 0 & 0 & 0.05930 \end{array}\right)\left(\begin{array}{c} X\\ Y\\ Z \end{array}\right)$$ (1)

Since the Macleod and Boynton diagram requires a decision about the absolute height of the S fundamental, we chose this free parameter (see last row in the 3x3 matrix in Eq 1) so that the StDev of our experimental results involving the achromatic (Gr) boundary with all other colours is the same for both chromatic axes when plotted in our new cone chromaticity space. This purely arbitrary normalization has the aim of simplifying our model fittings by making our data approximately isotropic. The LMS values were then transformed to chromatically-opponent *lsY* data using Eq 2:

$$\begin{array}{l} l=\frac{L}{L+M};\\ s=\frac{S}{L+M};\\ Y=L+M; \end{array}$$ (2)

According to Eqs 1 and 2, the values of *Y* corresponding to the CIE L*a*b* lightness planes we measured (L = [36, 47, 58, 76, 81, 86]) were Y = [11.23, 19.97, 32.37, 62.20, 72.90, 84.76] respectively.

The cone chromaticity space represented by Eq 1 is both convenient for our modelling (see below) and physiologically relevant since its axes, when normalized so that zero is the “white”, are coincident to those reported for cells in the LGN of macaque [2, 6, 102]. These cells, carrying both chromatic and luminance information from the retina correspond to the vast majority of the input to the striate cortex.

**Fig 5:**
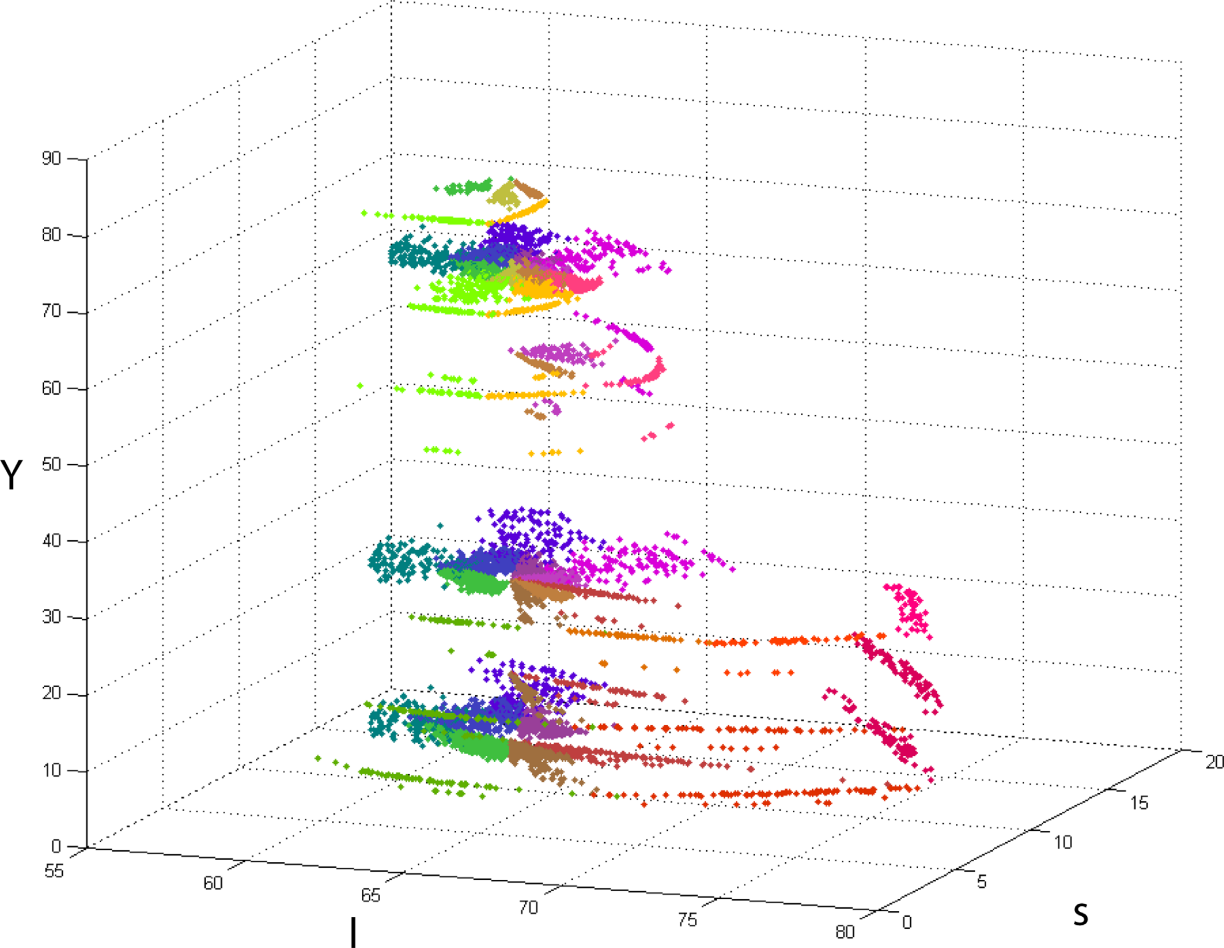
lsY plot of all experimental results (Conditions 1 and 2). The scale of the s-axis was chosen so that the StDev of the achromatic boundaries data is the same in both l and s directions (see last row in the 3x3 matrix in Eq 1).

Fig 5 shows an *lsY* plot of the same data presented in Fig 2. Colour-coded points represent the corresponding categorical boundary. As before, points’ colours have been chosen as the mathematical average of the two categories considered. The data is in broad agreement with the rest of the literature, e.g. focal points collected in different colour systems and under different illuminants [74, 76, 103] broadly fall within the limits defined by our categorical boundaries. In particular, the shapes and distributions of points in Fig 5 are consistent with the shapes of the elliptical distributions of consensus colours in the work of Cao *et al* [103] which were estimated for 10 deg eccentricity viewing.

### Parametric fittings to categorical boundary data in *lsY*

**Fig 6:**
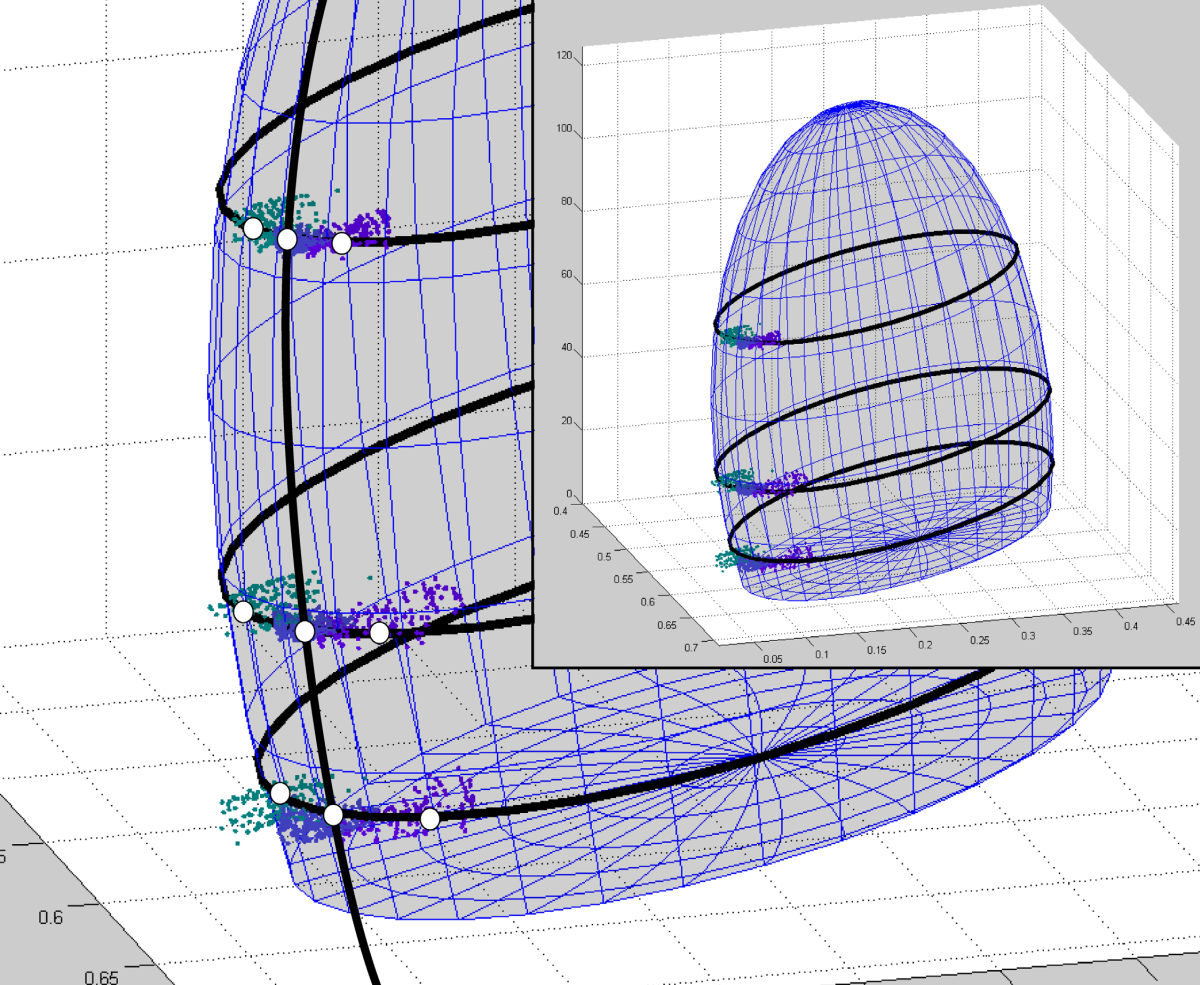
Details of an exemplary 3D-ellipsoid fitting to the psychophysical results irrespective of experimental condition. Data points correspond to the boundaries of “blue” with all its neighbours (green, purple and achromatic borders were considered). Black ellipses are the intersections between 3D-ellipsoid and the horizontal planes corresponding to the three Y-levels measured: ***Y***_***low***_ = 11.23, ***Y***_***med***_ = 32.37 and ***L***_***high***_ = 72.90. The A vertical ellipse was inserted to highlight the three points that are necessary to fully determine its curvature. White circles represent the weight exerted in the fit by data points related to each of the nine boundaries measured. All observers and conditions were considered.

The variability of the data allows for fitting several models. For example it is common to segment the chromatic space using fuzzy set regions [78, 81] or simply lines (planes) stemming from the achromatic locus towards more saturated regions [79]. In our case we settled for the simplicity of 3D ellipsoids for two main reasons: (1), the distributions of data points in Fig 5 strongly hint of elliptical curvatures in 3D and (2) ellipsoids are parsimonious shapes that depend on the squares of distances to a central point weighted by a parameter and correspond well to the non-linear computations of cortical neurons in ATD space [8]. According to this, the most convenient function to model our 3-dimensional *lsY* regions of categorical consensus, i.e. an ellipsoid of the form:

$${\left(\frac{l-l_{0}}{a}\right)}^{2}+{\left(\frac{s-s_{0}}{b}\right)}^{2}+{\left(\frac{Y-Y_{0}}{c}\right)}^{2}=1$$ (3)

which is defined by six parameters: centre [*l*_0_, *s*_0_, *Y*_0_], and semiaxes [a, b, c]. We also considered three parameters of rotation: *α*, *β*, *γ* the counterclockwise rotation angles around each of *lsY* axes respectively. When the 3D-ellipsoid defined by Eq 3 intersects with horizontal planes (constant-Y) it determines 2D-ellipses, which are similar in shape to the distributions of focal colours in Cao *et al* [103]. As an example, Fig 6 shows an ellipsoidal fit to the three *Y*-levels of “blue” categorical boundaries in *lsY* space. To fit the data we first centred all points by subtracting [*l*_0_, *s*_0_, *Y*_0_]. Next we applied a rotation matrix to all data points, generating rotations around the *l*-, *s*-, and *Y*-axes [104, 105], so that Eq 3 can be applied without adding any angular parameter. Following this, we calculated the shapes of the 3D-ellipsoids by minimising the residual sum of squares (RSS) of the distances between the ellipsoids and all points. The best fit was determined by simultaneously modifying the nine ellipsoid parameters. Our fits included all measurements, i.e. all observers, backgrounds and conditions and was produced using the constrained nonlinear multivariable search algorithm implemented by Matlab’s *fmincon* function [106]. In most cases, algorithm initialization was based on the consensus regions (focal colours) of Cao *et al* [103]. In other cases initialization was done manually. We imposed the criterion that final values should be between 10% and 200% of the original initialization value.

Using this method, we fitted eight colour ellipsoids, i.e. “green”, “blue”, “purple”, “pink”, “red”, “orange”, “yellow”, “brown”, and an “achromatic” ellipsoid. To be able to compare our results to others in the literature, we split the “achromatic” ellipsoid into three different categories (“grey”, “white” and “black”) by conducting an extra ad-hoc experiment. The achromatic ellipsoids share the same parameters for rotations, as well as centre and semiaxes lengths in *l* and *s* directions. They only differ by their parameters in the luminance direction (i.e. their position and length along the *Y* axis).

**Fig 7:**
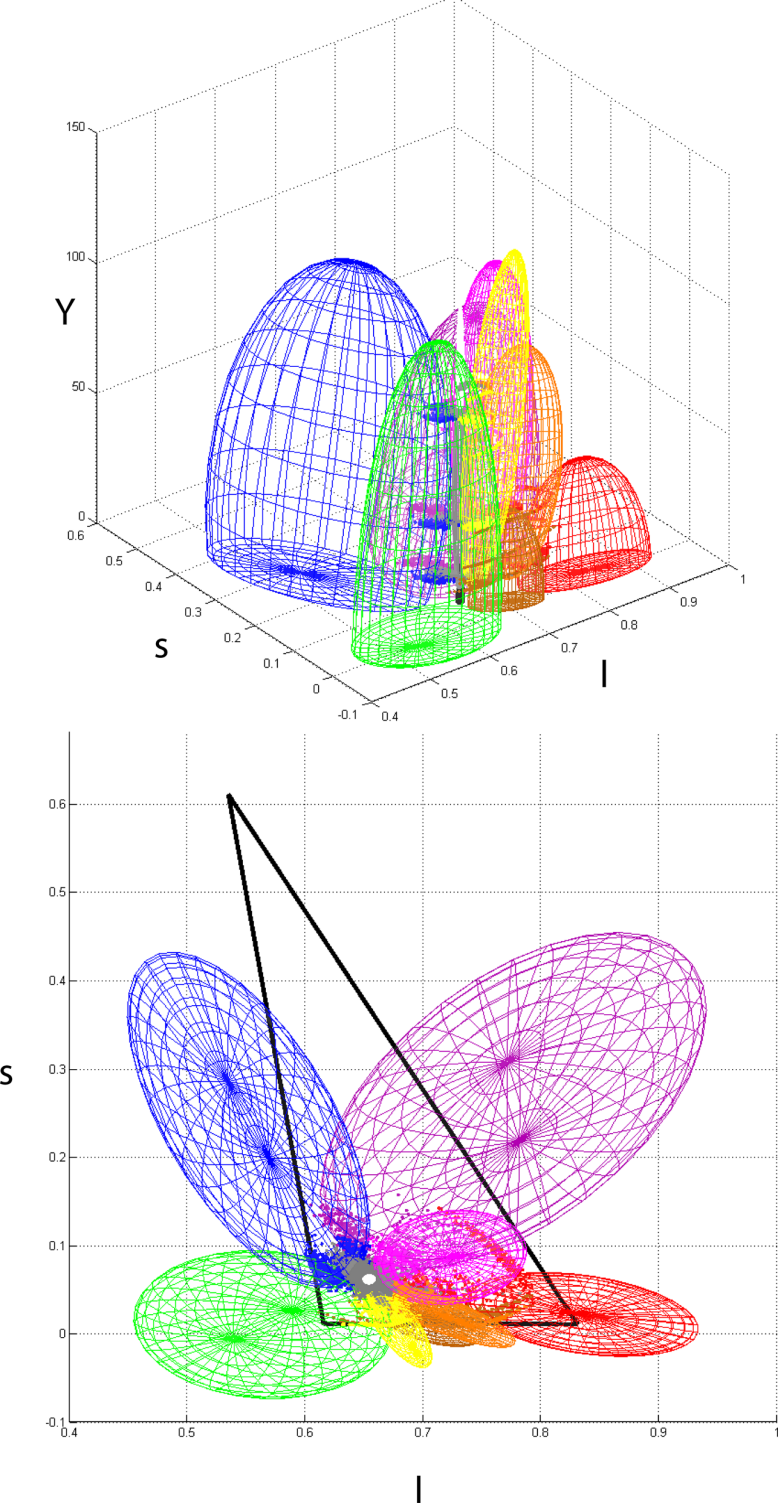
Side and top views of our 3D-ellipsoidal fittings to all categorical boundary data in *lsY* space. For each category, we considered all points bordering with its categorical neighbours, including the achromatic centre. The ellipsoids’ sizes and positions were calculated by minimizing the planar distances between the points and the ellipses generated by intersecting the 3D-ellipsoid with the six constant-Y (horizontal) planes described in the main text. The top view also includes the triangle formed by the monitor’s RGB phosphorous.

Fig 7 shows two different views of the ellipsoidal fittings to all psychophysical results. The variability of the data determines some volumetric overlap between neighbours, which is consistent with the variability exhibited by colour naming results in the literature (see introduction). The bottom plot includes an estimation of the gamut defined by the monitor’s phosphorous used in our experiment. Because we did not cover the whole range of visible colours in our experiments, parts of the solid determined by the monitor’s phosphors fall outside the ellipsoids. In particular, the region of highly saturated “purple” and “blue” which is largely determined by the “blue” monitor phosphorous is not well represented in the model. This region consists mostly of low intensity light and to include it, it might be necessary to extend our experiments to lightness ranges below the minimum considered here. Something similar might be said about the small region of highly saturated “purple” and “pink” which is not covered by our ellipsoids. Table 3 shows the results of fitting 3D-ellipsoids to all of our experimental results. Columns show the parameters that define the ellipsoids (centre, semiaxes and rotation angle in degrees counterclockwise).

**Table 3:** Results of fitting ellipsoids to the experimental results using the Nelder-Mead simplex direct search algorithm [106]. Columns refer to the ellipsoid centre and semiaxes, and rotation around each of the axes.

Category	Centre			Semiaxes			Rotation (deg)		
	l	s	Y	l	s	Y	l	s	Y
green	0.534	0.009	0	0.081	0.108	110.0	1.1	1.1	77.6
blue	0.552	0.241	35.1	0.075	0.198	90.0	2.9	0.0	22.0
purple	0.776	0.262	0.2	0.115	0.220	83.6	2.2	2.2	321.5
pink	0.723	0.085	66.5	0.066	0.053	55.5	0	0.6	17
red	0.836	0.021	0	0.099	0.044	44.0	0	1.1	168.0
orange	0.719	0.019	52.5	0.019	0.066	45.0	1.1	0	61.8
yellow	0.677	0.009	72.9	0.018	0.043	67.5	2.9	0	27.5
brown	0.703	0.019	0	0.027	0.054	36.0	0.6	0	62.7
grey	0.654	0.060	39.9	0.005	0.005	38.5	0	0	0
white	0.654	0.060	90	0.005	0.005	25.0	0	0	0
black	0.654	0.060	0	0.005	0.005	15.0	0	0	0

In our mechanistic model, an input image is processed by the precortical neural machinery, resulting in three ATD signals which then enter the cortex and activate isoresponsive neurons tuned to a limited portion of the chromatically-opponent space. The output of these neurons is gathered at a later stage by forward processing layers, resulting in a series of overall 3D “chromatic tuning” functions whose shape is based on the ellipsoids defined in Table 3. This “categorical belonging” is not just dualistically determined by whether a given input is in- or out-of-an-ellipsoid function, but it is subject to some degree of uncertainty, particularly at the categorical boundaries (as shown by our psychophysical results). We modelled this uncertainty as a 3-dimensional response function representing the probability of a given colour to be named as belonging to each of the categories represented by the ellipsoids of Table 3. As a result, every pixel in the input image is labelled with 11 numbers representing how much they belong to each category: pixels that fall well inside one of the ellipsoids will have a large “belonging value” (e.g. close to 1) for this category and close to 0 for the others and pixels that fall near a categorical boundary will have values close to 0.5 for the two neighbouring categories and almost 0 for the others. We modelled the large variability common to all categorization decision data using a non-linear logistic function defined by:

$$P={\left(1+\text{exp}(g(|x-c|-h)\right)}^{-1}$$ (4)

where *P* is in the interval [0, 1] and represents the degree of “belonging”, *c* is the centre of the 3D ellipsoid considered, *x* is the distance between c and the pixel considered, *g* is the growth ratio of the sigmoidal and *h* is the position of the half-height transition point. In practice *h* is equal to the distance from *c* to the 3D ellipsoid in the direction joining *c* and the pixel considered. When the point represented by x is inside the ellipsoid we have *h* > |*x-c*| then the factor g*(|*x-c*|-*h*) becomes negative and *exp*(g*(|*x-c*|-*h*)) tends to zero, which in turn makes the result of Eq 4 tend to 1 (maximum belonging). Other values of *x* produce smaller values of P. We arbitrarily set *g* to be equal to the average of the semi-axes of each ellipsoid, a choice that seems to agree well with the variability observed in the psychophysical results. This way larger ellipsoids (colour categories) have steeper boundaries and therefore the belongingness of pixels outside drops faster.

Eq 4 solves the practical problem of having regions of the chromatic space not encompassed by any of the categories defined in Table 3. This is a consequence of both having a limited number of categories and the stochastic component of colour categorization. Points that are “outside” or “in between” can still be categorized by Eq 4 with values that are smaller than unity. However, this solution gives rise to another problem: all pixels have non-zero “belonging values” for all categories, most of them extremely small. These represent the very small likelihood that, for example, a person assigns the name “red” to a blue patch. This likelihood can be manipulated using the growth ratio *g* of the model in Eq 4, but a more comprehensive solution will be to add more categorical regions in the future to fill the whole 3D space. The fact that belongingness (P-values) are never zero has to do with the continuous nature of the functions chosen to model our data. A thresholding of low values will solve this inconvenience (indeed making the model far less elegant) but a quick estimation of P-values shows this might not be necessary: the P-values of a pixel midway between the red and green centroids according to Eq 4 are P_R_ = 2.8·10^−05^ and P_G_ = 3.6·10^−06^. Similarly for a pixel midway between the yellow and blue centroids (falling inside the blue ellipsoid): P_Y_ = 1.5·10^−20^ and P_B_ = 1.0.

The fact that NICE is parametric allows the possibility of adjusting the shape and position of each ellipsoid to simulate the effects of corticogeniculate feedback. This feedback projects to the LGN in parallel streams that are likely to selectively modulate the responses of magnocellular, parvocellular and even koniocellular neurons. The functional role of V1 feedback is not clearly elucidated, but it is known to multiplicatively increase parvocellular responses and adjust the reliability, timing, and burst/tonic activity profile of LGN neurons in response to visual stimuli (for a review see Briggs and Usrey [107]). There is also growing evidence of behavioural/cognitive modulation on LGN neurons, e.g. visual spatial attention effects [108]. Since these cognitive (memory, attention, language, cultural background, etc.) effects may influence the results of a categorization task via feedback, we including these processes in our model by adjusting its properties to the psychophysical results of many observers.

### Ground-truth testing results

**Fig 8:**
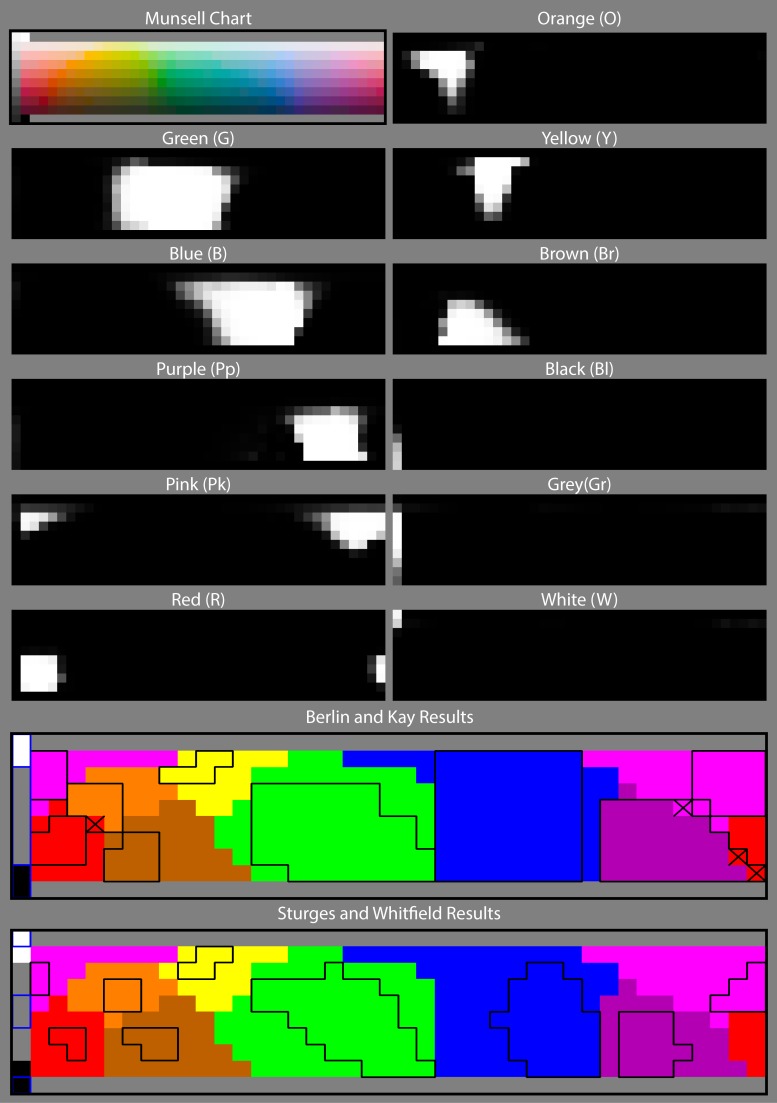
Results of our chromatic categorization model when applied to a simulated Munsell Chart. The first panel shows the original image and subsequent panels show the value of “belonging” (obtained from Eq 4) for each colour and category. The bottom panels show psychophysical results represented by black boxes (obtained by Berlin & Kay [27] and Sturges & Whitfield [74] respectively), superimposed to ours.

**Fig 9:**
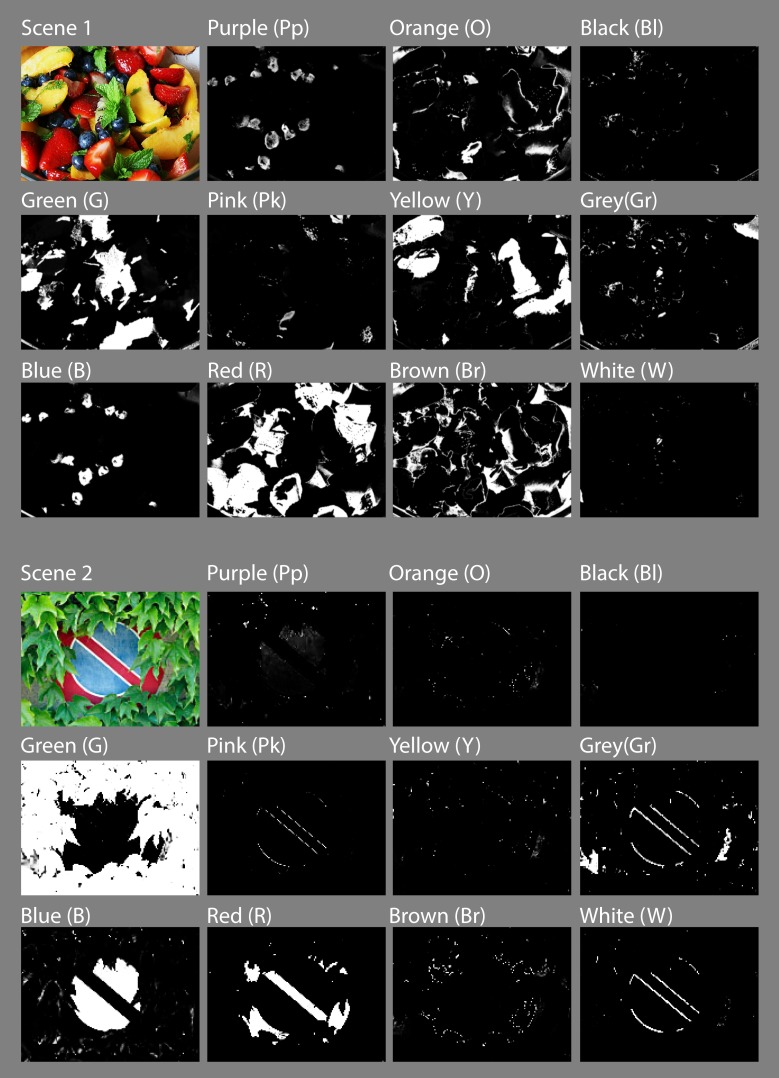
Typical chromatic categorization results of NICE shown for two different natural scenes. Coloured panels show the original image and subsequent panels show the value of “P” for each colour and category.

Fig 8 shows NICE’s results for a simulated Munsell Colour Chart [109]. The top left panel shows the original chart and subsequent panels show the value of P in greylevels for the different categories including the separation of Grey into Grey (Gr), Black (Bl) and White (W). The two bottom panels show the psychophysical segmentation of the same chart as published by Berlin & Kay [27] and Sturges & Whitfield [74] (black boxes). NICE’s results are superimposed over the boxes in colour and show a near complete agreement with both sets of results everywhere except in the squares marked with an “x”. We also run NICE on a number of natural scenes with some striking results. Fig 9 shows an exemplary set of results for two such natural scenes.

## Discussion

A complete understanding of colour vision entails a complete understanding of how signals from the three cone photoreceptors are combined throughout the visual pathway and ultimately the cortex to produce the strikingly rich perception of colour that we possess. Regarding the first stages, it is well established that the neural mechanism sensitive to red-green variation (the parvocellular- or P- pathway) originates in the retina and conveys signals through the LGN to the cortex [2]. Less well known is the substrate behind the complementary mechanism sensitive to yellow-blue variation (that has been associated to the koniocellular or K-pathway), which is likely to receive substantial input from S-cones. This input has been shown to diverge in many important respects according to whether it is excitatory or inhibitory, e.g. excitatory S-inputs cells have more homogeneous spatiochromatic properties than inhibitory S-inputs [13]. These asymmetries have been linked to LGN measurements and behavioural work [6, 9]. Further up in the pathway, the lack of agreement between the cardinal directions measured at the LGN level [2] and those revealed psychophysically [9] and at the level of the cortex [6–8] suggests the existence of further non-linear transformations, some of which have been modelled by adding a perceptual opponent stage beyond the already known cone opponent stage [10, 11]. Indeed the existence of a broad distribution of chromatic responses among cortical neurons suggests that in addition to the cardinal mechanisms, many less prominent mechanisms (tuned to various directions in colour space) are present in the cortex [6, 7].

In the case of chromatic categorization, there is no agreed theory of how the brain achieves this prompt and unique identification and categorization of colours. Although language exerts a dominant and complex influence, psychophysical evidence hints at the presence of common low-level, perceptual components behind same/different decisions in both categorization and discrimination tasks [38]. This perceptually-based machinery linking the mechanisms responsible of chromatic discrimination to those behind colour naming is certainly subject to modulatory feedback from higher cortical areas carrying the effects of cognition and language. This feedback is most likely introduced at the level of the LGN which impacts over a large population of V1 neurons and whose input includes higher areas of the brain (e.g. visual cortex, superior colliculus, and parts not directly involved in visual perception) [107, 108, 110]. We chose not to enforce these higher-order chromatic mechanisms explicitly but to rather fit our model to psychophysical data within the framework provided by the chromatic signatures of the thalamocortical pathway [2]

Since the aim of the present study is to contribute to bridge the gap between the behavioural and neurophysiological approaches, we started by defining the overall properties that a chromatic categorization system should have to operate in the colour space determined by the responses of opponent and non-opponent cells at the level of the LGN. To do so we psychophysically measured the boundaries between nine universal chromatic categories defined as 3D regions in the ATD space of MacLeod and Boynton [97]. Following this we found the *most parsimonious* mathematical representation of these regions in the form of 3D ellipsoids which are good descriptions of the categorical boundaries emerging from our psychophysical results. It turns out that a large proportion of visual cortex neurons are equipped with the computational machinery to respond to stimuli arranged in ellipsoidal 3D surfaces such as the ones that best describe our categorical regions cortex [8]. Based on these facts we hypothesized that these visual cortex neurons may be responsible for the first stages of chromatic categorization and propose a model as a *proof of concept*, i.e. a demonstration of the feasibility of this theory. The model incorporates psychophysical and neurophysiological evidence of chromatic computation present in the retina and LGN [1–3, 11, 101], and uses these signals as input to a hypothetical recreation of the non-linear processing present in the cortical machinery [8, 21–24]. The later part is highly conjectural, since much of the evidence shows that colour processing in the cortex is not related to colour perception in any obvious way [14–16, 111]. However, it has been argued [8] that these inconclusive results are the product of the inadequacy of linear characterization methods and that V1 neurons combine signals in a non-linear but systematic way, with isoresponses in chromatically opponent space that are either planar, cup-shaped or ellipsoidal. These 3D-figures were modelled from a collection of points that evoked the same firing rate in V1 neurons when presented with drifting Gabor patterns. The same study shows that about half of the neurons measured exhibited ellipsoidal responses, with preferences for axes that were aligned to perceptually-identified colour directions. Although the neurons described by Horwitz and Hass were tested using stimuli centred on the achromatic locus, their results and model show how curved isoresponse surfaces can be constructed by nonlinearly combining signals from linear neurons. In this line, we modelled the responses of colour categorization neurons based on similar nonlinear combinations: our ellipsoids are built using the same kind of neural computations modelled by Horwitz and Hass.

Our study differs from previous psychophysical studies in two important ways: (1) we evaluated the distribution of colour categories in 3D space, i.e. considering variations in lightness and (2) we focused our measurements in the chromatic boundaries instead of focal colours. This experimental method for identifying boundaries is better than previous methods since it concentrates the data collection in regions of maximum uncertainty. Had we employed the most common approach of measuring regions of agreement we would have obtained unreliable boundaries (obtained mostly by averaging distances among pairs of focals) with little information about inter-observer variability at the boundaries. Furthermore, knowing the shape of the boundaries gave us a clear advantage when it came to fit unknown 3D-shapes to the psychophysical data, however there were still many possible solutions that could fit the data and we decided for the simplest: 3D-ellipsoids. Other solutions (such as Gaussians, triple sigmoidals, or even vertical planes) can also fit our data in 3D however, the complexity and number of parameters make them very difficult to justify from a biological point. 3D ellipsoids need only 10 parameters to fit (i.e. we need to measure three boundaries at three different lightness levels to define them) and their shapes resemble the shapes that evoke identical firing rate in isoresponsive neurons in the visual cortex.

Although the current model configuration is based on a limited set of psychophysical results, its predictions can be generalised by accommodating other categorical regions and boundaries such as olive, turquoise, skin, peach, etc. This is very straightforward and only requires considering pixels with intermediate values (e.g. a pixel with both P_Green_ and P_Blue_ <1) as belonging to a transitional category. Another feasible solution is to add more ellipsoids by conducting further psychophysical measurements. Additionally, ellipsoidal shapes are extremely versatile from a modelling point of view: their shapes and positions can be easily adjusted in future iterations of the model to take into account overall as well as local image characteristics, adapting them to variations in illumination and surround contrast. This in turn could lead to insights about categorical "illusions", such as the identical X categorized as yellow or grey according to the background (see Fig 5.9 in [90]).

Having a versatile modelling solution has many advantages. For example, by fitting our ellipsoids to the averaged results of many observers we are able model the (cognition- and language- based) higher-level influences that are apparent in our categorization data. Indeed, our psychophysical results show that inter-observer variability is smaller than the variability from individual observers (see Table 2). This remarkable agreement across colour-normal observers (at least for the particular task and colour boundaries tested here) supports the claim that some kind of cortical normalization operates over the chromatic mechanisms [9, 60], promoting the invariance of colour categories across observers which in turn favours a more accurate verbal communication. These results (in addition to the mismatch between physiological- and psychophysically-measured cardinal axes [6]) can be explained by the presence of cortical feedback at the level of the LGN [107]. The success of NICE with standard colour charts and naturalistic stimuli verifies that our approach although incomplete and tentative, has the potential of being correct.

Additional testing shows that our quantitative modelling results are slightly better than those of current state-of-the-art chromatic categorization algorithms. Table 4 shows the compared results of several colour categorization models when applied to both the Berlin & Kay and Sturges & Whitfield psychophysical results. These models are: Lammens’s Gaussian model (LGM) [112]; MacLaury’s English Speaker (MES) [113]; Benavente and Vanrell’s Triple Sigmoid model (TSM) [114]; Seaborn’s fuzzy k-means model (SFKM) [85]; Benavente *et al*’s Triple Sigmoid- Eliptic Sigmoid model (TSMES) [81]; van de Weijer *et al*’s Probabilistic Latent Semantic Analysis (PLSA) [87] and ours.

**Table 4:** Comparative results of several colour categorization models when applied to the Berlin & Kay [27] and Sturges & Whitfield [74] psychophysical results. The data for LGM, MES, TSM, SFKM and TSEM was obtained from Table 2 in Benavente *et al* [81].

	Berlin and Kay results			Sturges and Whitfield results		
	Coincidences	Errors	% errors	Coincidences	Errors	% errors
LGM	161	49	23.33	92	19	17.12
MES	182	28	13.33	107	4	3.6
TSM	185	25	11.9	108	3	2.7
SFKM	193	17	8.1	111	0	0
TSEM	193	17	8.1	111	0	0
PLSA	187	23	12.3	109	2	1.8
**NICE**	**206**	**4**	**1.9**	**111**	**0**	**0**

As a further test, we ran NICE on the psychophysical results of Hansen *et al* [79] whose observers had to categorize 417 coloured patches as belonging to one of eight categories (red, orange, yellow, green, turquoise, blue, purple, and grey). Our results came slightly worse than before (7.7% of errors) mainly because of the many differences between the paradigms used to collect Hansen’s data and ours, namely their viewing conditions were different; they ignored chromatic regions such as pink and brown and added an intermediate region (turquoise) not present in our paradigm. Moreover, their subjects (native German speakers) had to select the category to which colour samples belong in an experiment conducted in German. This higher-level language influence is likely to have contributed to the larger number of mismatches. However, despite of this our results show that our low-level model is robust enough to account for results in both the computer vision and psychophysics literature which include some top-down, language contributions. As a comparison, results from other models (TSEM and PLSA) are also included in Table 5.

**Table 5:** Comparative results of three state-of-the-art colour categorization models when applied to the Hansen *et al* [79] psychophysical results. All observers, colour samples and viewing conditions were considered for the neutral illumination condition.

Hansen *et al* results			
	Coincidences	Errors	%errors
TSEM	4423	197	4.3
PLSA	4339	281	6.1
**NICE**	**4265**	**355**	**7.7**

We have found that in contrast to previous studies where subjects had to memorize best examples of colours [115], our observers had great difficulty in reproducing the same boundary even after substantial training. As discussed in the introduction there are many sources of variability which affect our data, widening the shape of the histograms in Fig 3 and Fig 4. We account for this variability by including a sigmoidal term in Eq 4, since our objective is to study and model the low-level side of the phenomenon which is arguably common to all humans. However, from Figs 3 and 4 it is clear that this slope parameter is not the same for all ellipsoids and boundaries and can be adjusted to consider each ellipsoid’s variability separately in future versions of the model. We have also tested for systematic effects such as to whether the presence of a variegated or flat background may influence categorization and our results show this not to be the case for the present paradigm. We believe our measurement error (associated to the method of adjustments) plus the large observer variability is likely to mask smaller effects such as those observed by Brown and MacLeod.

The question of whether the sensory information at the level of ATD mechanisms forms the basis of categorical perception has not been settled in the literature. Some researchers postulated that our basic ability to discriminate colours should fully explain why we use a particular set of categories to communicate about colours. In this line Regier *et al* found sensitivity for colour differences to be higher across than within category boundaries in CIEL*a*b* space [116], but Witzel and Gegenfurtner [69] only found a loose relationship between colour categorization and discrimination after measuring discrimination thresholds and colour categories around an isoluminant hue. Other experiments found that reaction times did not follow named colour categories in visual search [117]. However we would argue that these results only challenge the idea of a *direct link* between category effects and sensitivity to colour differences. In our model, we postulate that the precise location of a categorical boundary is a non-linear function of second-stage mechanisms, is subject to neural noise and varies from observer to observer (and even from one trial to the next). This accumulation of variability and non-linearity explains why it is so difficult to prove the existence of a link between continuous colour perception and colour categorization by exploring category effects.

At its core, the model is built around the hypothesis that there is a collection of neurons equally-sensitive to colour stimuli organized in the shape of ellipsoids in opponent colour space. In this view each neuron categorizes a small portion of the space by firing when a stimulus falls within the volume defined by this ellipsoid. Subsequent layers build over the outputs of the previous so that the overall effect resembles the large ellipsoids found psychophysically and recreated by NICE. Such an arrangement would benefit from neurons sensitive to the same chromatic stimuli to be located physically close. Although there is ample evidence of such mechanisms in the cortical processing of spatial frequency (e.g. the spatial contrast sensitivity function appears to be the envelope of many narrowly tuned SF selective channels), there is no evidence of similar arrangements for chromatic categorization. However, the discovery of V1 neurons that functionally perform these seemingly complex operations and the discovery of chromatically selective clusters in this and other areas support the ideas proposed here. In summary, our work does not intend to provide a complete, quantitative and rigorous testing of our ideas but rather a proof of concept, a demonstration of feasibility which contributes to show their potential for future use.

## Conclusions

Our work attempts to tackle the explanatory gap between colour perception and colour naming by providing a biologically-plausible model of chromatic categorization. Since visual signals enter the cortex in the form of two chromatically opponent and a luminance channel, we started by measuring the boundaries of chromatic categories in an ATD-colour space derived from cone photoreceptor sensitivities and postreceptoral processing mechanisms. To this end, we defined a psychophysical experiment explicitly targeted to measure categorical boundaries and explored nine of the universal categories originally proposed by Berlin and Kay [27]. We parsimoniously modelled these nine regions as 3D ellipsoids, which are similar to the shapes that evoke identical firing rate in isoresponsive neurons in the visual cortex [8]. Finally we tested our simple model with results at the same level or above the state-of-the-art.

Although NICE is by no means a finished product, our results are surprisingly encouraging for such a simplistic setup. Each categorical region is basically defined by weighted sums of squared cone-contrast and luminance inputs and needs only few parameters to be specified. It is difficult to imagine a simpler system to segment colour space into categories, which is arguably a point on its favour. For all these reasons we believe in the plausibility of this model from a biological point of view.
